# Supplementary material for: Targeted Inhibition of O-Linked β-N-Acetylglucosamine Transferase as a Promising Therapeutic Strategy to Restore Chemosensitivity and Attenuate Aggressive Tumor Traits in Chemoresistant Urothelial Carcinoma of the Bladder
Source: Biomedicines. 2022 May 18;10(5):1162. doi: 10.3390/biomedicines10051162 (PMC9138654; doi:10.3390/biomedicines10051162)

Supplementary Figure S1. The validation of OGT gene-silencing with shRNA in UMUC-3 and T24 cells. UMUC-3 and T24 cells stably expressing shCTL or shOGT were incubated for 48 h and OGT expression was examined by WB and qRT-PCR. The data are representative of at least three independent experiments. Error bars indicate the mean ± SEM for three independent experiments. ***P < 0.001.

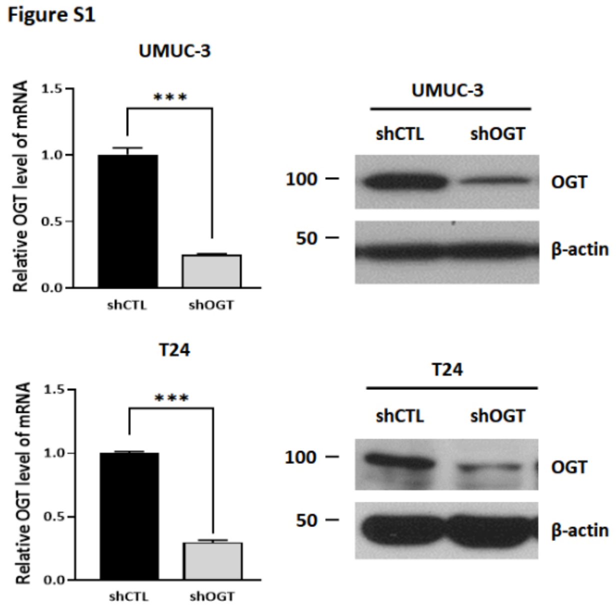

Supplementary Figure S2. Comparison of cell morphology between chemoresistant clones and their parental cells. Representative phase-contrast microscopic images demonstrating morphology of parental and matched chemoresistant sublines, magnification 200X.

Figure S2

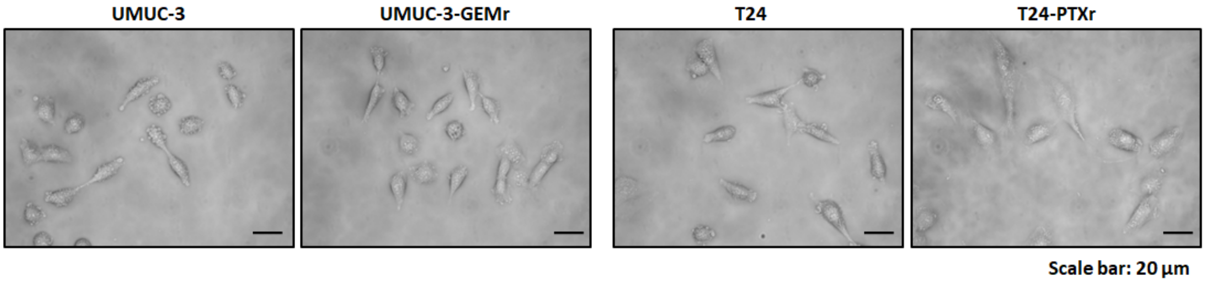

Supplementary Figure S3. Comparison of cell growth kinetics between chemo-resistant clones and their parental cells. The cells were incubated in the IncuCyte™ analyzer to monitor doubling time. Cell confluence levels of these cells were measured real-time and presented as a percentage using the IncuCyte™ analyzer. The data are representative of at least three independent experiments. Error bars indicate the mean ± SEM for three independent experiments.

Figure S3

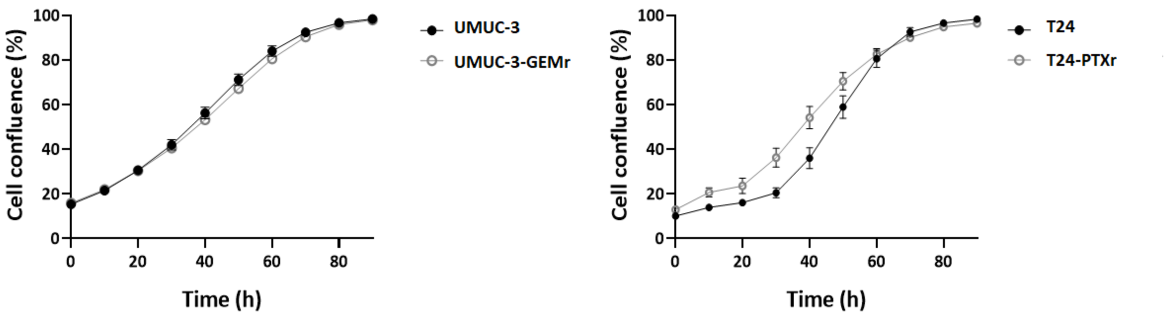

Supplement: Supplementary file 1 [file biomedicines-10-01162-s001.zip › biomedicines-10-01162-s001/biomedicines-1717535-supplementary.pdf]
